# The Synergy of ADAM17-Induced Myocardial Inflammation and Metabolic Lipids Dysregulation During Acute Stress: New Pathophysiologic Insights Into Takotsubo Cardiomyopathy

**DOI:** 10.3389/fcvm.2021.696413

**Published:** 2021-06-04

**Authors:** Joseph Adu-Amankwaah, Gabriel Komla Adzika, Adebayo Oluwafemi Adekunle, Marie Louise Ndzie Noah, Richard Mprah, Aisha Bushi, Nazma Akhter, Yaxin Xu, Fei Huang, Benard Chatambarara, Hong Sun

**Affiliations:** ^1^Department of Physiology, Xuzhou Medical University, Xuzhou, China; ^2^Department of Medicine, Xuzhou Medical University, Xuzhou, China

**Keywords:** takotsubo cardiomyopathy, pathophysiology, ADAM17, acute myocardial inflammation, metabolic lipids dysregulation, acute stress, therapeutic targets

## Abstract

Due to its reversible nature, Takotsubo cardiomyopathy (TTC) is considered an intriguing and fascinating cardiovascular disease characterized by a transient wall motion abnormality of the left ventricle, affecting more than one coronary artery territory, often in a circumferential apical distribution. Takotsubo cardiomyopathy was discovered by a Japanese cardiovascular expert and classified as acquired primary cardiomyopathy by the American Heart Association (AHA) in 1990 and 2006, respectively. Regardless of the extensive research efforts, its pathophysiology is still unclear; therefore, there are no well-established guidelines specifically for treating and managing TTC patients. Increasing evidence suggests that sympatho-adrenergic stimulation is strongly associated with the pathogenesis of this disease. Under acute stressful conditions, the hyperstimulation of beta-adrenergic receptors (β-ARs) resulting from excessive release of catecholamines induces intracellular kinases capable of phosphorylating and activating “A Disintegrin and Metalloprotease 17” (ADAM17), a type-I transmembrane protease that plays a central role in acute myocardial inflammation and metabolic lipids dysregulation which are the main hallmarks of TTC. However, our understanding of this is limited; hence this concise review provides a comprehensive insight into the key role of ADAM17 in acute myocardial inflammation and metabolic lipids dysregulation during acute stress. Also, how the synergy of ADAM17-induced acute inflammation and lipids dysregulation causes TTC is explained. Finally, potential therapeutic targets for TTC are also discussed.

## Introduction

Takotsubo cardiomyopathy (TTC) is an acute, stress-induced cardiac syndrome characterized by a transient wall motion abnormality of the left ventricle, affecting more than one coronary artery territory, often in a circumferential apical distribution (1). This condition is also known as; stress cardiomyopathy, ampulla cardiomyopathy, stress-induced cardiomyopathy, apical ballooning cardiomyopathy, transient left ventricular dysfunction, Gebrochenes-Herz-syndrome, and broken heart syndrome (2). Takotsubo cardiomyopathy was first described in 1990 by a Japanese cardiovascular expert (3). In 2006, it was classified as acquired primary cardiomyopathy by the American Heart Association (AHA) (4). It is initiated by extreme physical or emotional stress and can occur in females and males at any age; however, postmenopausal females are commonly affected. Takotsubo cardiomyopathy can also be caused by infections, such as severe acute respiratory syndrome coronavirus 2 (SARS-CoV-2) (5, 6). The diagnosis of this condition is generally based on clinical criteria combined with a multi-modality imaging approach which includes coronary angiography (with left ventriculography), electrocardiography (ECG), cardiac magnetic resonance imaging (CMR), and transthoracic echocardiography (TTE) (7). Takotsubo cardiomyopathy is usually reversible within a few weeks; hence, its prognosis was initially considered favorable (8). Nevertheless, research suggests that the long-term prognosis of TTC is poorer than anticipated (8) since it accounts for substantial morbidity and mortality worldwide (9). Regardless of the extensive research efforts, its pathophysiology is still not clear (8). Due to this, there are no well-established guidelines specifically for treating and managing TTC patients. It is, therefore, vital to understand the pathomechanisms that enhance the development of TTC.

Sympatho-adrenergic stimulation is strongly associated with the pathogenesis of TTC (10, 11). Indisputably, the histological alterations in the myocardium during TTC are comparable to those found in catecholamine-induced cardiotoxicity in humans and animals (12). Current studies have also revealed that acute myocardial inflammation and metabolic lipids dysregulation are implicated in the pathogenesis of TTC (9, 13). However, how sympatho-adrenergic stimulation leads to TTC characterized by the latter is still unclear.

Intriguingly, “A Disintegrin and Metalloprotease 17” (ADAM17) forming part of the 560 proteases encoded in the human genome has recently emerged as a key regulator of inflammation and lipids dysregulation following stressful conditions. “A Disintegrin and Metalloprotease 17” is ubiquitously expressed in human tissues, including the brain, heart, kidney, and skeletal muscle (14, 15). Although ADAM17's expression is downregulated in physiological states, several studies have demonstrated its upregulation along with its substrates [tumor necrosis factor-alpha (TNFα) and soluble interleukin-6 receptor (sIL-6R)] in dilated cardiomyopathies (16–19). Hence, this indicates ADAM17's essential role in the etiology of TTC. Besides ADAM17 inducing cardiomyopathies *via* exacerbating inflammatory responses and lipids dysregulation, its variants and mutants have been associated with mild cardiomyopathies and congenital heart defects, including Tetralogy of Fallot (20, 21).

In acute stress state, the hyperstimulation of beta-adrenergic receptors (β-ARs) due to excessive release of catecholamines from sympathetic responses exert negative inotropic and chronotropic effects on the heart (11), thereby increasing the activities of intracellular kinases such as receptor-stimulated p38 mitogen-activated-protein-kinases (p38 MAPKs) and extracellular signal-regulated kinases (ERKs) (22, 23). These kinases are well-known activators of ADAM17 *via* their intracellular phosphorylation (24). Hyperactive ADAM17 plays a crucial role in acute cardiac inflammation (17) and myocardial lipids dysregulation (9), which successfully results in left ventricular abnormalities characterizing TTC. However, our understanding of this is limited; hence this concise review provides a comprehensive insight into the key role of ADAM17 in acute myocardial inflammation and metabolic lipids dysregulation during acute stress. Also, how the synergy of ADAM17-induced acute inflammation and lipids dysregulation causes TTC is explained. Finally, potential therapeutic targets for TTC are discussed.

## Activation of ADAM17 During Acute Stress

Acute stress has been recognized as a modifiable risk factor for several cardiomyopathies (25). The impact of acute stress on physiological and psychological processes is determined by the stress stimulus's characteristics, either emotional or physical. However, in both, the autonomic nervous system is one of the central neural pathways activated (26). Thus, in an acute stress state, the sympathetic system's hyperstimulation results in elevated catecholamines (epinephrine and norepinephrine). Physiologically, epinephrine and norepinephrine serve as neurotransmitters and hormones necessary for homeostasis maintenance; however, excessive increase in their levels leads to the hyperactivation of β-ARs, which are the main receptors mediating the inotropic and chronotropic function of the heart (9, 23). Beta-adrenergic receptors are 7-transmembrane, G-protein coupled receptors (GPCRs) which exist in three subtypes, namely; β1-AR, β2-AR, β3-AR (27, 28). All are widely expressed in the heart, with β1-AR having the highest expression and β3-AR with the least expression (23, 27). β1-AR can only signal through Gαs when activated while β2-AR and β3-AR can signal through Gαs or Gαi upon activation depending on the condition (physiological or pathological) (23, 29). Physiologically, upon activation, β2-AR signals through Gαs (23) while β3-AR signals through Gαi (29).

**Figure 1:**
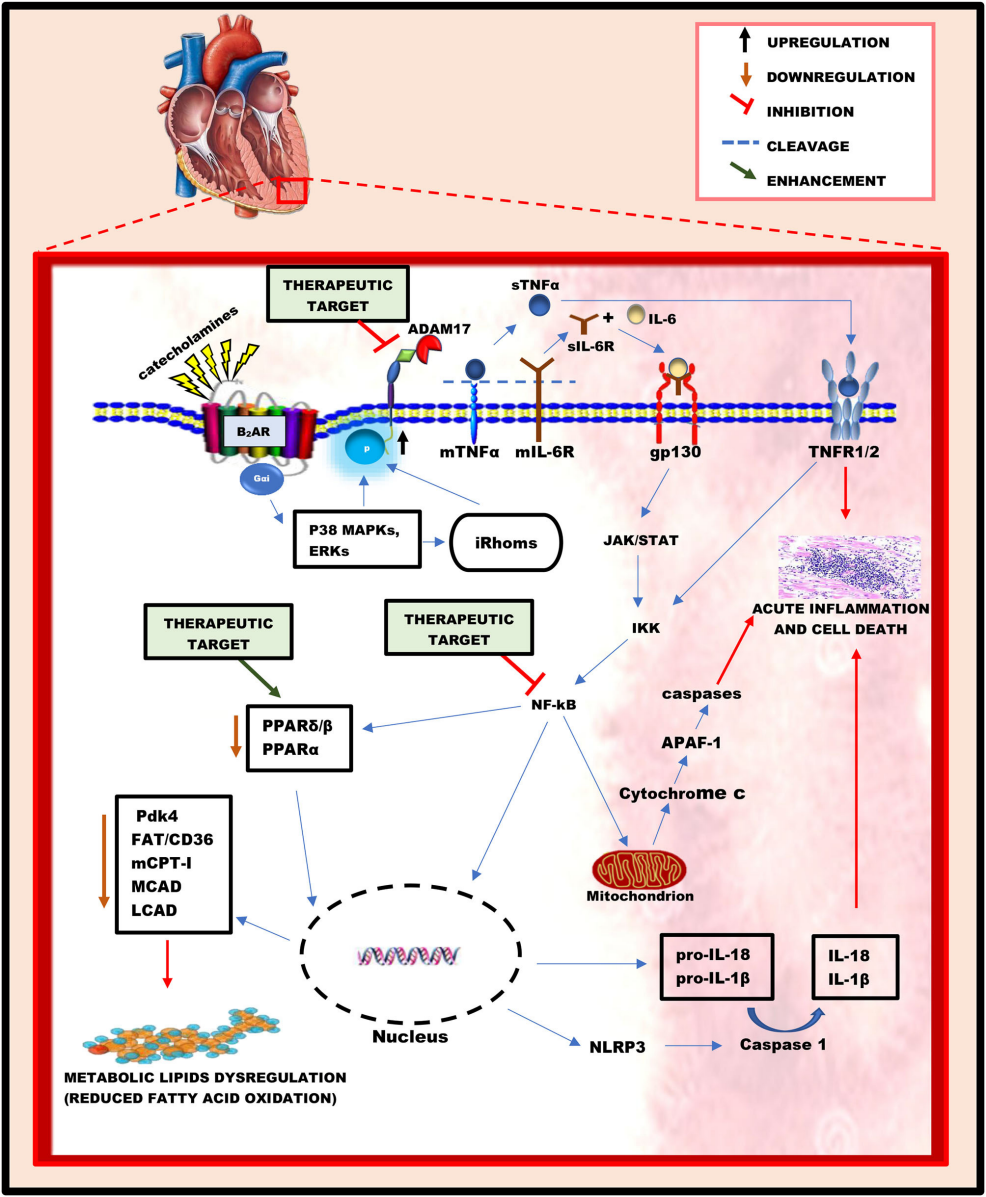
A schematic diagram illustrating the key roles played by ADAM17 and its substrates in inducing acute myocardial inflammation and metabolic lipids dysregulation during acute stressful conditions. In an acute stress state, the hyperactivation of beta-adrenergic receptors resulting from the excessive release of catecholamines influences the activation of ADAM17 *via* intracellular phosphorylation by ERKs and p38 MAPKs. Hyperactive ADAM17 cleaves and activates TNFα and IL-6R to trigger cell signaling cascades, leading to NF-kB activation. Active NF-kB successfully causes acute myocardial inflammation *via* the gene elevation of pro-inflammatory cytokines (IL-18, IL-1β) and inflammasomes (NLRP3) in the nucleus, as well as the release of cytochrome c from the mitochondria. Additionally, the activation of NF-kB causes a reduction in the activity of PPARβ/δ and PPARα, thereby leading to a decreased mRNA and protein levels of key regulatory enzymes of fatty acid oxidation, which characterizes metabolic lipids dysregulation. Hence, the proposed therapeutic targets for the attenuation of acute myocardial inflammation and metabolic lipids dysregulation may include; the inhibition of ADAM17 and NF-kB's activation and enhancing the activities of PPARβ/δ and PPARα.

Hyperstimulation of β-ARs under acute stressful conditions, characterized by an excessive increase in catecholamines, causes desensitization of β1-AR and hyperactivation of β2-AR coupling to Gαi. This occurs because, among the three β-AR subtypes, β2-AR and β3-AR are rarely depleted during acute stress (30, 31). To prevent cardiac injury caused by acute stress, Gαi signaling *via* the non-canonical pathway increases the activities of intracellular kinases such as p38 MAPKs and ERKs (22, 23). Interestingly, studies have revealed that p38 MAPKs and ERKs can positively influence the activation of ADAM17 *via* their intracellular phosphorylation, either directly or indirectly, through the activation of iRhoms (32, 33). Physiologically, ADAM17's expression can be regulated by transcriptional factors such as nuclear factor kappa B (NF-kB) and ETS Like-1 (Elk-1) (34). However, post-transcriptional mechanisms such as chromatin remodeling protein BRG1 affect its expression. Also, subcellular localization in the perinuclear region has been shown to regulate the activities of ADAM17 (35). Nonetheless, phosphorylation of threonine 735 in ADAM17 by active kinases can rapidly activate its cleavage activity, possibly by triggering and facilitating its translocation from the Golgi network to the cell surface where its proteolytic activity is reported (36, 37). Structurally, ADAM17 comprises a prodomain, a catalytic domain, a disintegrin-like domain, a membrane-proximal domain (MPD), and a short stalk region, which together form the extracellular part of the protease and are connected to an intracellular region by a transmembrane part (38). The stalk region of this protease contains the CANDIS motif (Conserved ADAM 17 Dynamic Interaction Sequence), which is found closer to the MPD around the plasma membrane and is vital for substrate recognition. (39). Intracellular phosphorylation of ADAM17 induces the phosphatidylserine exposure at the outer leaflet of the cell membrane, causing it to bind to the membrane through its MPD and CANDIS, thereby initiating its activation and cleaving process (40). Upon activation, ADAM17 cleaves and stimulates pro-inflammatory cytokines and their cognate receptors (14, 15, 41, 42), resulting in acute inflammation and metabolic lipids dysregulation in the heart (Figure 1).

## ADAM17 in Acute Myocardial Inflammation and Lipids Dysregulation

### Activation and Signaling of ADAM17's Substrates

“A Disintegrin and Metalloprotease 17” has emerged as a chief regulatory hub in inflammation due to cleavage and activation of several pro-inflammatory cytokines and their cognate receptors. The most noticeable examples include; TNFα, tumor necrosis receptor 1 and 2 (TNFR1 and 2), and IL-6R (14, 15, 41, 42). These cytokines are widely expressed in the heart as membrane-bound proteins (43). Excessive increase in their soluble forms following the cleavage process of ADAM17 can trigger cascades resulting in acute cardiac inflammation, myocardial lipotoxicity, and low energy production.

Soluble TNFα (sTNFα), when released under acute stressful conditions, can signal *via* TNFR1 or TNFR2 (44, 45), which are predominantly expressed on myocytes (44). Interestingly, soluble TNFRs (sTNFR1/2) resulting from ADAM17's cleavage process can also bind to membrane TNFα (mTNFα) and act as antagonistic decoy receptors known as “reverse signaling,” which is seen chiefly among other TNF family members (46, 47). Interleukin-6 (IL-6) is a pleiotropic cytokine with distinct pro-and anti-inflammatory properties when released under stressful conditions. It can signal *via* two different ways, namely; (1) Signaling through its membrane-bound receptor, IL-6R, which is known as “classic signaling” and can only occur on cell types expressing surface IL-6R, including hepatocytes and certain leukocytes' subpopulations (48). (2) Signaling *via* the soluble form of its receptor, sIL-6R, which is termed as “IL-6 trans-signaling” and can occur on all body cells, including cardiomyocytes, since the IL-6/sIL-6R complex can directly bind to and activate the ubiquitously expressed glycoprotein-130 (gp130) without the need of a membrane-bound IL-6R (43, 48) (Figure 1).

### ADAM17's Substrates in Acute Myocardial Inflammation and Lipids Dysregulation

The binding of sTNFα and IL-6/sIL-6R complex to TNFR1/2 and gp130, respectively, can trigger intracellular signaling cascades leading to acute myocardial inflammation (49, 50) and metabolic lipids dysregulation (9, 51, 52). The intracellular region of TNFR1 contains a death domain that can directly induce apoptosis and inflammation when activated (43). However, this domain is absent in the intracellular region of TNFR2. The activation of TNFR2 by sTNFα mediates the phosphorylation of the p65 subunit of NF-Kb at ser536 *via* interaction with the IkB kinase (IKK), subsequently resulting in the activation of NF-kB dimer (52, 53). Similarly, the binding of the IL-6/sIL-6R complex to gp130 is capable of inducing phosphorylation and activation of NF-kB dimer *via* gp130/JAK/STAT pathway (49). Following activation, NF-kB migrates into the mitochondria (54, 55) and nucleus (56) to induce signaling cascades and up/down-regulate genes, respectively. According to Liu et al., NF-kB can stimulate the intrinsic apoptotic pathway in the mitochondria *via* the release of cytochrome c (50). In the cytoplasm, cytochrome c binds to apoptotic protease activating factor 1 (APAF-1), causing it to undergo conformational changes and oligomerization into a heptameric wheel-like structure known as apoptosome, which recruits and activates the initiator caspase-9 (57). Active caspase-9 cleaves and activates the executioner caspases-3 and −7, resulting in rapid apoptosis and inflammation in cardiac cells (50, 57). Additionally, in the nucleus, active NF-kB upregulates genes of pro-inflammatory cytokines (pro-IL-18 and pro-IL-1β) and NLR family pyrin domain containing 3 (NLRP3), hence elevating their protein levels. NLRP3 is an intracellular sensor that can be triggered under acute stressful conditions, resulting in the formation and activation of NLRP3 inflammasome (58, 59). Active NLRP3 inflammasome is known to activate caspase 1, which in turn cleaves pro-IL-1β and pro- IL-18 to release their soluble forms, successively inducing necrosis and inflammation in cardiac cells (60) (Figure 1).

Interestingly, studies have also revealed that the activation of NF-kB downregulates the activity of peroxisome proliferator-activated receptor (PPAR)β/δ, α (51, 52) and genes regulating fatty acid (FA) oxidation in the heart (52). Peroxisome proliferator-activated receptors (PPARs) are a group of nuclear receptors that serve as transcription factors regulating the expression of metabolic genes within cells (61). PPARs comprises three subtypes, namely; PPARα, PPARγ, and PPARβ/δ (61). Fatty acids are well-known endogenous ligands of the PPAR family (62). PPARβ/δ and PPARα are widely expressed on cardiac cells, which serve as transcriptional regulators of myocardial energy and lipid homeostasis (51). According to Planavila et al., activation of NF-kB caused a reduction in the expression of pyruvate dehydrogenase kinase 4 (Pdk4), a target gene of PPARβ/δ, which plays a vital role in fatty acid utilization and palmitate oxidation. The reduction in the activity of PPARβ/δ was proposed to be caused by the physical interaction between the p65 subunit of NF-kB and PPAR β/δ during the phosphorylation and activation of NF-kB (51). Also, a study carried out by Pellieux et al., revealed that the activation of NF-kB is associated with the downregulation of PPARα's activity (52). Physiologically, the activation of PPARα upregulates the mRNA and proteins levels of key regulatory enzymes of fatty acid oxidation, hence reduction in its activity due to the activation of NF-kB decreases the levels of these key enzymes, which includes; fatty acid translocase/cluster of differentiation 36 (FAT/CD36), carnitine palmitoyltransferase I (mCPT-I), medium-chain acyl-CoA dehydrogenase (MCAD), and long-chain-acyl-CoA dehydrogenase (LCAD) (52, 63, 64). Fatty acid enters the cell *via* FA transporters on the cell membrane, including FAT/CD36 (65). Within the cell, the conversion of long-chain fatty acyl CoA to an acylcarnitine required for entry into the mitochondria is carried out by mCPT-I (62). In the mitochondria, MCAD and LCAD play vital roles in converting FA into energy during β-oxidation (62, 66). Thus, these enzymes play crucial roles in the uptake and utilization of FA. In short, activation of NF-kB subsequently causes a reduction in FA oxidation *via* downregulation of FA oxidation pathways. The decrease of FA oxidation in the heart is accompanied by intramyocardial lipid accumulation and reduced myocardial energy, characterizing myocardial lipids dysregulation (9) (Figure 1).

## The Synergy of ADAM17-Induced Acute Inflammation and Lipids Dysregulation in TTC

**Figure 2:**
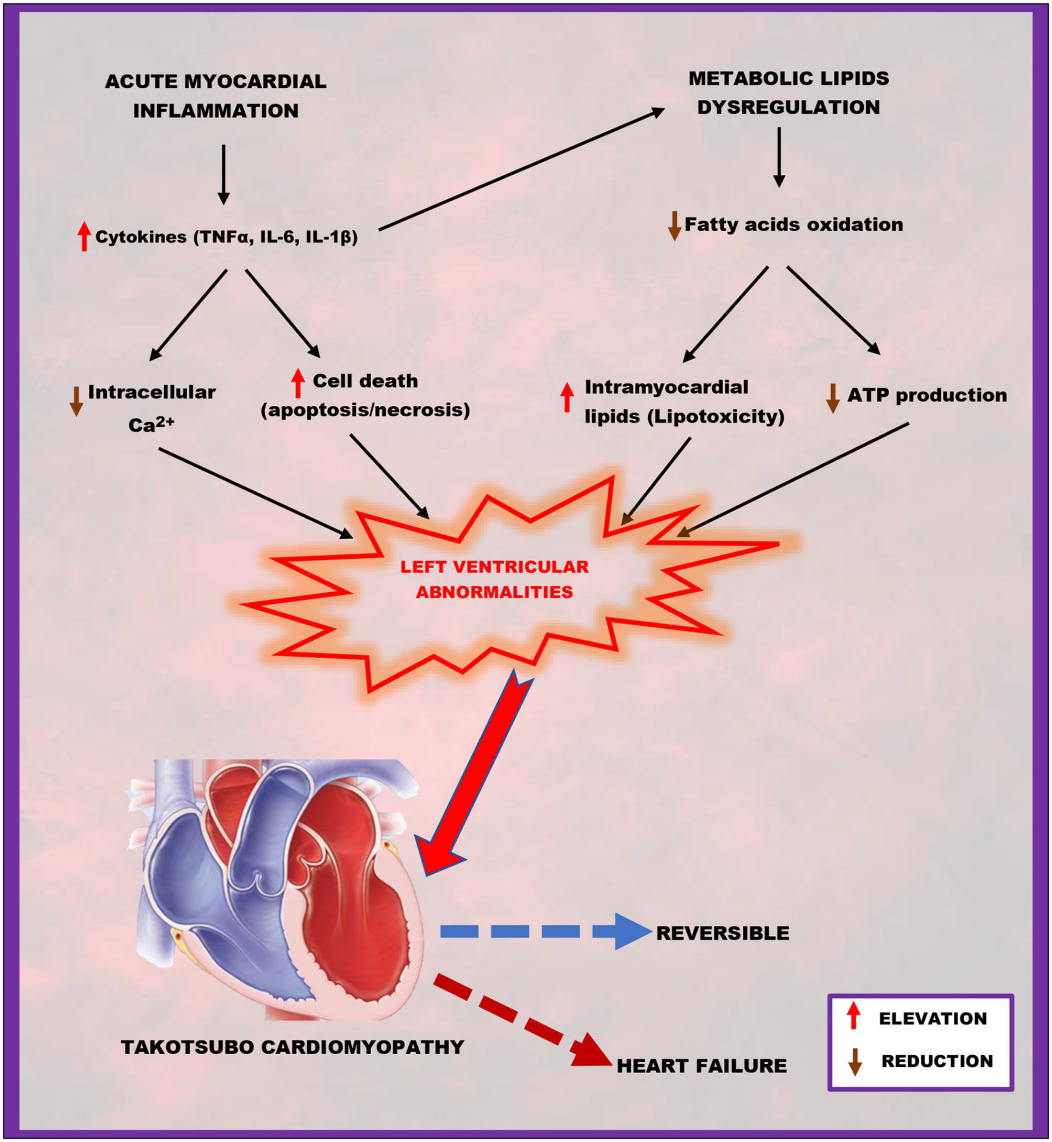
A schematic representation of how acute myocardial inflammation, in combination with metabolic lipid dysregulation, contributes to takotsubo cardiomyopathy. Elevated cytokines (TNFα, IL-6, IL-1β) and reduced fatty acid oxidation resulting from ADAM17-induced acute myocardial inflammation and metabolic lipids dysregulation successfully lead to left ventricular abnormalities characterized by reduced intracellular calcium and adenosine triphosphate (ATP) production; increased cell death (apoptosis/necrosis) and intramyocardial lipids accumulation (lipotoxicity), which are the main hallmarks of takotsubo cardiomyopathy.

Studies have shown that acute myocardial inflammation (13) and lipotoxicity (9) are implicated in the pathogenesis of TTC. It is well-known that cytokines released during inflammation exert detrimental effects on the heart. Thus, cytokines like TNF-α, IL-6, and IL-1ß cause cardiac necrosis and apoptosis (60) and downregulate the expression of calcium (Ca^2+^)-regulating genes, including sarcoplasmic reticulum Ca^2+^ ATPase (67) and Ca^2+^-release channel (68), resulting in a direct negative inotropic effect on the heart (69, 70). It is well-established that Ca^2+^ ions are responsible for the electrical activation and mechanical contraction of the the myocardia (71), hence reduction in intracellular Ca^2+^ coupling with cell death following acute inflammation can lead to abnormalities in the contraction of the left ventricle. Also, ADAM17-induced lipid dysregulation under acute stress state is accompanied by intramyocardial lipid accumulation and reduced myocardial production of adenosine triphosphate (ATP) (9). Excessive accumulation of lipids in the myocardia increases levels of toxic intermediates leading to lipotoxicity (72). Low ATP production and lipotoxicity may affect left ventricular function *via* abnormal cardiac contraction development (72). In a nutshell, acute myocardial inflammation coupling with lipids dysregulation triggered by active ADAM17 under acute stressful events may be the main inducers of the transient wall motion abnormalities of the left ventricle characterizing TTC, which is usually reversible after few days or may progress to heart failure (Figure 2).

## Therapeutic Targets for TTC

As yet, there are no well-established therapeutic guidelines specifically for treating TTC patients (73); however, based on the broad knowledge gained from this concise review, potential therapeutic targets for the treatment and management of TTC might lie in the direct inhibition of ADAM17 and NF-kB or indirectly antagonizing their activation and activities. The most promising therapeutic inhibitors of ADAM17 includes tissue inhibitor of metalloproteinase 3 (TIMP3), protein disulfide isomerases (PDIs), and integrins (43). Remarkably, direct injection of TIMP3 in the heart has been shown to downregulate ADAM17's activity by binding to its catalytic domain, thereby inactivating it (74, 75). Also, PDIs can directly interact with the MPD of ADAM17, where it catalyzes the isomerization of two disulfide bridges, thus downregulating ADAM17's activity (76, 77). A study conducted by Bax et al., revealed that the binding of intergrin α_5_β_1_ to ADAM17 *via* its disintegrin domain, downregulated its activity by affecting its mediated cell adhesion and migration (78). Aside from its natural inhibitors, a variety of miRNAs, including miR-145 (79), miR-124 (80), miR-152 (81), and miR-326 (82), have been proven to downregulate ADAM17's expression and reduce its substrate release by directly binding to the ADAM17 3′-UTR. Regarding NF-kB, there are several approaches to inhibiting its transduction pathway, including receptor inhibition, adaptor inhibition, IKK inhibition, IkB stabilization, cytoplasmic retention, and transcription factor inhibition (83). NF-kB inhibitors have been grouped into three categories: biomolecular inhibitors, synthetic chemicals, and natural products (and their derivatives) (83). Biomolecular inhibitors include siRNAs, decoy oligonucleotides (containing the kB site), ribozymes, the IkB super-repressor, interfering peptides, and dominant-negative molecules (83). Most synthetic chemicals are molecules engineered against components of the IKK complex, which plays a central role in NF-kB activation (84, 85). Natural products include a wide range of marine-, plant-, and microbe-derived compounds that target different steps in the NF-kB pathway (86–88). These natural products are categorized into three main groups; IKK inhibitors, antioxidants, and thiol-reactive compounds that can target several stages of the NF-kB signaling pathway. Among 19 drugs reported in previous studies, digitoxin, ectinascidin 743, ouabain, chromomycin A3, and bortezomib were the most potent NF-kB inhibitors (89). The inhibition of ADAM17 and NF-kB is key in attenuating acute myocardial inflammation and lipids dysregulation, which are the hallmarks of TTC (Figure 1).

Additionally, the usage of pharmacologic agents capable of enhancing the activities of PPARβ/δ and PPARα might be a promising target for the treatment and management of TTC by preventing the reduction of key regulatory proteins of FA oxidation *via* two different mechanisms (52). The first mechanism involves direct activation of the transcription of target genes by binding to peroxisome proliferator responsive elements in the promoter region (52). The second mechanism is a direct interaction with transcription factors involved in the hypertrophic response, including NF-kB and activator protein 1 (AP-1), which may, directly or indirectly, contribute to reducing the proteins of FA oxidation (90). Studies suggest that the activity of PPARα can be enhanced by fibrate hypolipidemic drugs (91, 92). However, there are currently no commercially available drugs capable of enhancing the function of PPARβ/δ (92). The maintenance of optimal levels of key regulatory proteins of FA oxidation is vital in ameliorating cardiac function *via* the attenuation of lipotoxicity and increasing myocardial ATP production (Figure 1).

## Conclusions and Future Perspectives

Although TTC is considered a unique and interesting cardiomyopathy due to its reversible nature, it is still underappreciated among clinicians and researchers. Several promising pathophysiologic theories have been suggested, but this condition's exact underlying mechanistic processes remain unclear. This concise review has provided some general insights into this disease's pathogenesis; however, considering the prevalence in postmenopausal women, TTC could be linked to the modulation of acute myocardial inflammation and metabolic lipids dysregulation by sex hormones and the endocrine system at large. Thus, much remains to be discovered about TTC. Hence many areas require further exploration to understand this multifaceted cardiomyopathy perfectly.

## Author Contributions

The review idea was conceived by JA-A. JA-A drafted and wrote the manuscript with the supervision of HS. JA-A, GA, AA, MN, RM, AB, NA, FH, YX, and BC revised and proofread the manuscript. All authors contributed to the article and approved the submitted version.

## Conflict of Interest

The authors declare that the research was conducted in the absence of any commercial or financial relationships that could be construed as a potential conflict of interest.
